# Expression and Function of Allergin-1 on Human Primary Mast Cells

**DOI:** 10.1371/journal.pone.0076160

**Published:** 2013-10-07

**Authors:** Kei Nagai, Satoko Tahara-Hanaoka, Yuko Morishima, Takahiro Tokunaga, Yoshimasa Imoto, Emiko Noguchi, Kazumasa Kanemaru, Masamichi Imai, Shiro Shibayama, Nobuyuki Hizawa, Shigeharu Fujieda, Kunihiro Yamagata, Akira Shibuya

**Affiliations:** 1 Department of Immunology, Faculty of Medicine, University of Tsukuba, Tsukuba, Japan; 2 Department of Nephrology, Faculty of Medicine, University of Tsukuba, Tsukuba, Japan; 3 Department of Pulmonary Medicine, Faculty of Medicine, University of Tsukuba, Tsukuba, Japan; 4 Department of Medical Genetics, Faculty of Medicine, University of Tsukuba, Tsukuba, Japan; 5 Japan Science and Technology Agency (JST), Core Research for Evolutional Science and Technology (CREST), Tokyo, Japan; 6 Department of Otorhinolaryngology and Head and Neck Surgery, University of Fukui, Fukui, Japan; 7 Exploratory Research Laboratories, Tsukuba Research Institute, Ono Pharmaceutical Co., Ltd., Tsukuba, Japan; Osaka University, Japan

## Abstract

Mast cells (MC) play an important role in allergic and non-allergic immune responses. Activation of human MC is modulated by several cell surface inhibitory receptors, including recently identified Allergin-1 expressed on both human and mouse MC. Although Allergin-1 suppresses IgE-mediated, mast cell–dependent anaphylaxis in mice, the expression profile and function of Allergin-1 on human primary MC remains undetermined. Here, we established a seven-color flow cytometry method for assessing expression and function of a very small number of human primary MC. We show that Allergin-1S1, a splicing isoform of Allergin-1, is predominantly expressed on human primary MC in both bronchoalveolar lavage (BAL) fluid and nasal scratching specimens. Moreover, Allergin-1S1 inhibits IgE-mediated activation from human primary MC in BAL fluid. These results indicate that Allergin-1 on human primary MC exhibits similar characteristics as mouse Allergin-1 in the expression profile and function.

## Introduction

Mast cells (MC) are widely distributed throughout vascularized tissues, particularly near surfaces exposed to the external environment such as the skin, airways, and gastrointestinal tract. MC are well positioned to be involved in the first line of immune responses against environmental antigens, toxins, or invading pathogens[1]. MC express FcεRI, a high-affinity receptor for IgE, on their surface and play a central role in IgE-associated allergic responses [2]–[4]. Crosslinking of FcεRI-bound IgE with multivalent antigen initiates the activation of MC by promoting the aggregation of FcεRI, resulting in the degranulation of MC, along with the concomitant secretion of chemical mediators such as histamine, tryptase, carboxypeptidase A, and proteoglycans that are stored in the cytoplasmic granules of these cells, and the de-novo synthesis of pro-inflammatory lipid mediators such as prostaglandins and leukotrienes as well as platelet-activating factor in the early phase. MC also play an important role in innate immune responses against bacteria and parasites through the synthesis and secretion of cytokines and chemokines that recruit neutrophils, eosinophils, and Th2 cells to the site of infection [1], [5].

A major problem in MC research is the difficulty of obtaining primary MC, particularly in human, because MC are found not in the peripheral blood but in the systemic organs. Moreover, MC show a very low frequency in the systemic organs. Therefore, most MC experiments are performed with cultured MC derived from human blood progenitors or mouse bone marrow progenitors. However, the phenotypical and functional characteristics of MC depend on many factors, including species of animal, specific anatomical location, and status of maturation [6]. For example, although MC express IL-3 receptor, CD14, and Toll-like receptors in mouse, these molecules are scarcely detected on human MC [7]. The results of studies utilizing mouse MC are not directly transferable to human MC research. Therefore, it is desirable to be able to analyze human primary MC for research into allergic and non-allergic diseases mediated by MC effector function.

Activation of human MC is modulated by several cell surface inhibitory receptors, including FcγRIIB [8], SIRP-α·[9], CD300A (CMRF35) [10]–[12], and LILR-B2 [13]. We recently identified a novel immunoglobulin (Ig)-like inhibitory receptor, designated Allergy-inhibitory receptor (Allergin)-1, which contains the immunoreceptor tyrosine-based inhibitory motif (ITIM) in the cytoplasmic portion, in both human and mouse MC [14]. Mice deficient in Allergin-1 show significantly enhanced passive systemic and cutaneous anaphylaxis [14], indicating that Allergin-1 suppresses IgE-mediated, mast cell–dependent anaphylaxis in mice. Although mouse Allergin-1 contains one Ig-like domain in the extracellular portion, we identified three splicing soforms of human Allergin-1: Allergin-1 long form (Allergin-1L) that contains two Ig-like domains, Allergin-1 short-form 1 (Allergin-1S1) that contains the first Ig-like domain of Allergin-1L, and Allergin-1 short-form 2 (Allergin-1S2) that contains the second Ig-like domain of Allergin-1L in the extracellular portion. However, the expression profile of the Allergin-1 isoforms on human primary MC remains undetermined. Moreover, it remains unclear whether Allergin-1 inhibits the IgE-mediated activation of human primary MC.

In this study, we utilized flow cytometric method to assess the expression and function of Allergin-1 on a small number of human primary MC at a single cell level in bronchoalveolar lavage fluid (BALF) and nasal scratching specimens (NSS). We show that Allergin-1S1 is the major isoform expressed on human primary MC and that it inhibits IgE-mediated activation.

## Methods

### Samples

BALF was obtained from patients with pulmonary diseases at the University of Tsukuba Hospital, Japan. NSS were obtained from patients with non-seasonal allergic rhinitis at the Fukui University Hospital, Japan, and from healthy volunteers (**Table S1**). Peripheral blood and cord blood for the generation of cultured MC were obtained from healthy volunteers and RIKEN BioResource Center (Tsukuba, Japan), respectively. Written informed consent was obtained from the patients and healthy volunteers. This study was approved by the ethical review boards of both the University of Tsukuba and the University of Fukui. BAL was performed by using 150-mL aliquots of isotonic saline. NSS were obtained by brushing the unilateral inferior turbinates with a CytoSoft Cytology Brush (Medical Packaging, Camarillo, CA). BALF and NSS with blood contamination due to traumatic bleeding were excluded from the study. BALF and NSS were filtered through a 70-µm cell-strainer and resuspended in phosphate-buffered saline containing 2% fetal bovine serum.

### Antibodies, reagents, and transfectants

Human IL-3, IL-6, and Stem Cell Factor were purchased from PeproTech (London, UK). Monoclonal antibodies (mAbs) used in the flow cytometry analyses are shown in **Table S2**. TNP-specific mouse IgE was purchased from BD Biosciences (San Jose, CA). Anti-CD300A mAb (clone: TX49) was generated in our laboratory [15]. Anti-Allergin-1 mAbs (clones: EX29, EX32, and EX33) were provided by Exploratory Research Laboratories, Tsukuba Research Institute, Ono Pharmaceutical Co., Ltd., Japan. BW5147 cells stably expressing Allergin-1L, Allergin-1S1, or Allergin-1S2 were established by using a retroviral vector, as described [14].

### Flow cytometric analyses

For phenotypical analysis of primary MC, the cells obtained from the BALF or NSS were stained with propidium iodide (PI); Horizon V500–conjugated anti-CD45; a Horizon V450–conjugated mAb cocktail containing anti-CD3, anti-CD19, anti-CD56, anti-CD11b, and anti-CD11c mAbs; PE-Cy7–conjugated anti-c-Kit; FITC-conjugated anti-FcεRIα; and PE and/or APC-conjugated mAbs of interest (**Table S2**). Finally, cells were analyzed by using a FACS Fortessa with either Diva (Becton Dickinson, San Jose, CA) or Flow-Jo software (Tree Star, Inc. Ashland, OR). We purified cells by using a FACS Aria (Becton Dickinson, San Jose, CA) for morphological assessment.

### MC activation assay

After filtration of BALF, the cells were washed with HEPES-Tyrode's Buffer and then incubated with human IgG at 100 µg/mL in HEPES-Tyrode's Buffer to block nonspecific binding to Fcγ receptors. Cells were then incubated with 2 µg/mL trinitrophenol (TNP)-specific mouse IgE (Becton Dickinson) at 37°C for 2 h. After washing, cells were incubated with 200 µL of HEPES-Tyrode's Buffer containing various amounts of the TNP-conjugated F(ab′)_2_ fragment of the mAb against Allergin-1L and Allergin-S1 (clone: EX33) or the TNP-conjugated F(ab′)_2_ fragment of rat IgG (TNP-control Ig) at 37°C for 30 min, followed by incubation at 4°C for 10 min. Cells were then stained with APC-conjugated anti-CD107a, in addition to PI and the mAbs described above, to determine the MC population. Activation assay for Cord blood–derived cultured MC (CB-MC) and peripheral blood–derived cultured MC (PB-MC), cultured MC were incubated with 2 µg/mL TNP-specific mouse IgE containing culture medium at 37°C in 5% CO_2_ incubator for five days. Cells were then stimulated by 200 µL of HEPES-Tyrode's Buffer containing TNP-conjugated F(ab′)_2_ fragment of EX33 or TNP-control Ig at 37°C for 30 min, followed by incubation at 4°C for 10 min as well as BALF cells. After stimulation, cells were stained with APC-conjugated anti-CD107a, in addition to PI and PE-Cy7–conjugated anti-c-Kit to determine the MC population.

### MC culture

PB-MC and CB-MC were generated as described [16]. In brief, CD34^+^ cells were isolated from human peripheral blood mononuclear cells or cord blood mononuclear cells by using MACS cell separation system (Miltenyi Biotech) and cultured in serum-free Iscove's modified Dulbecco medium containing 80% methylcellulose medium, Stem Cell Factor (200 ng/mL), IL-6 (50 ng/mL), and IL-3 (1 ng/mL) for 6 to 8 weeks. Methylcellulose medium was then dissolved in phosphate-buffered saline, and the cells were cultured in Iscove's modified Dulbecco medium with 5% fetal bovine serum containing Stem Cell Factor (100 ng/mL) and IL-6 (50 ng/mL). For the phenotypical and functional analyses, PB-MC and CB-MC that had been cultured for more than 8 weeks were used.

### Quantitative RT-PCR analysis

**Figure 1:**
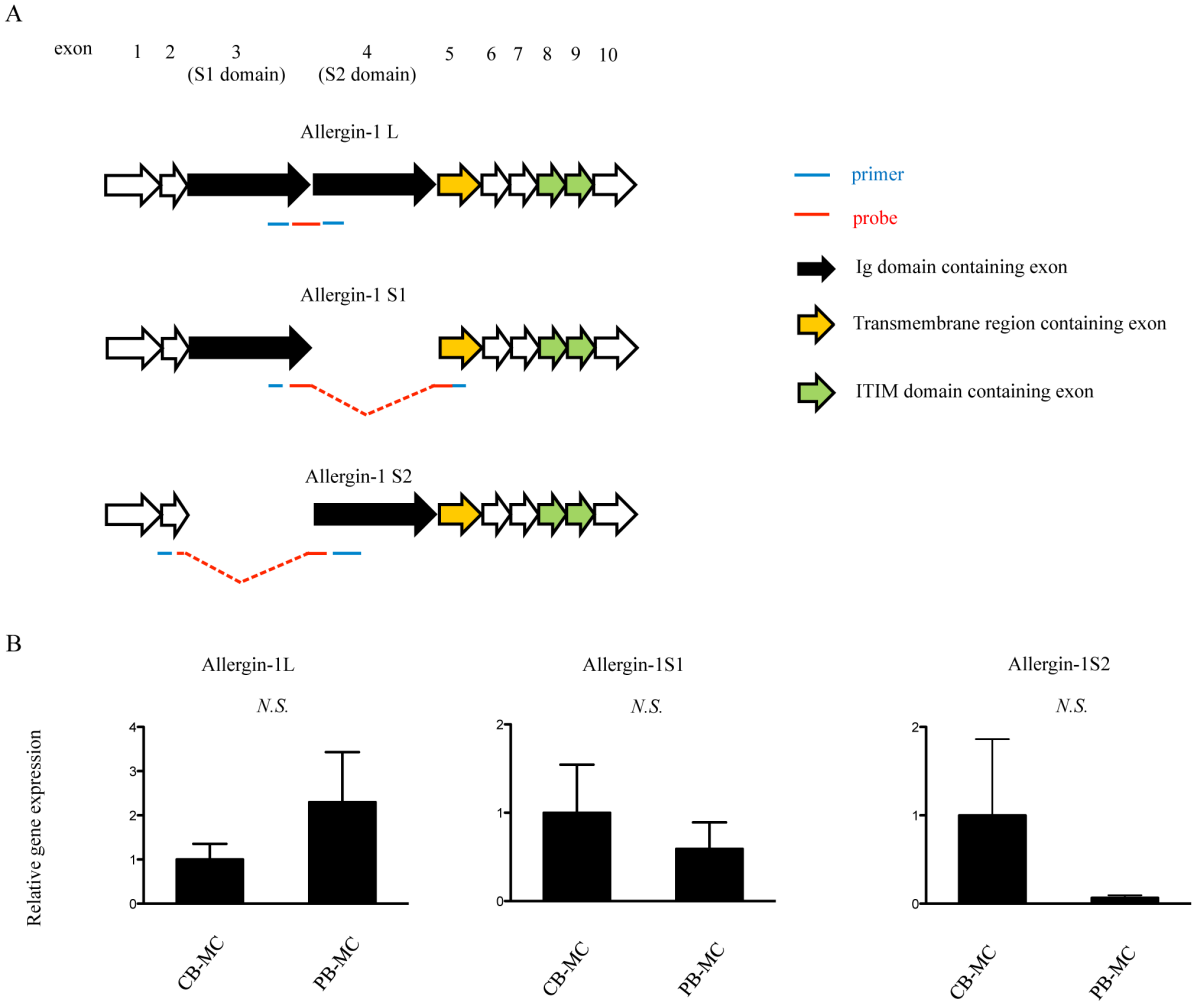
Expression of Allergin-1 isoform transcripts in cultured MC. A. Primers and probes for qRT-PCR were designed to detect Allergin-1L, Allergin-1S1 or Allergin-1S2 mRNAs. B. CB-MC (n = 3) and PB-MC (n = 3) cultured for more than eight weeks were subjected to qRT-PCR, as described in Method.

Total RNAs of CB-MC and PB-MC were isolated by ISOGEN (Nippon Gene), and the cDNA was synthesized with The High Capacity cDNA Reverse Transcription Kit (Invitrogen). For quantitative RT-PCR analysis, cDNA fragments were amplified with the TaqMan Universal Master Mix (Applied Biosystem) and quantified with the TaqMan Gene Expression assay (Applied Biosystem). The primers and probes were designed as shown **Fig. 1A**. The sequences were; 

Allergin-1L-Forward;5′-CCCAAGTTACCAGCTGTTCAAA-3′, 

Allergin-1L-Reverse;5′-TATATGTCGGTCTGTTTCTGTTTGAAT-3′, 

Allergin-1L-Probe;5′-CGATTGTCGACCCGGTG-3′, 

Allergin-1S1-Forward;5′-TAGGATTATCACCACCAGCAACAG-3′, 

Allergin-1S1-Reverse;5′-AGCTGTATTGGATTGTGAGGCA-3′, 

Allergin-1S1-Probe;5′-TCCGCCGACAATC-3′, 

Allergin-1S2-Forward;5′-AAATGCAAAGCCCAAGTTACCA-3′, 

Allergin-1S2-Reverse;5′-AATTGATGGGCAGCGAGC-3′, 

Allergin-1S2-Probe;5′-ACAAATGACCCGGTGACT-3′

### Statistical analysis

All values in the figures and text are expressed as mean ± SEM. Statistical significance of the differences among median values was evaluated by using the Mann–Whitney *U* test or Kruskal–Wallis one-way analysis with post-hoc Dunn's test to compare.

## Results

### Expression of Allergin-1 isoforms on human cultured MC

Unlike mouse Allergin-1, human Allergin-1 consists of three splicing isoforms: Allergin-1L, Allergin-1S1, and Allergin-1S2 (**Fig. 1A**) [14]. To analyze the expression profile of the Allergin-1 isoform in human MC, we performed PCR by using primer pairs that specifically detect the mRNA of each isoform (**Fig. 1A**). We detected all the isoforms in both CB-MC and PB-MC but observed no significant difference in the amount of each isoform between CB-MC and PB-MC (**Fig. 1B**).

**Figure 2:**
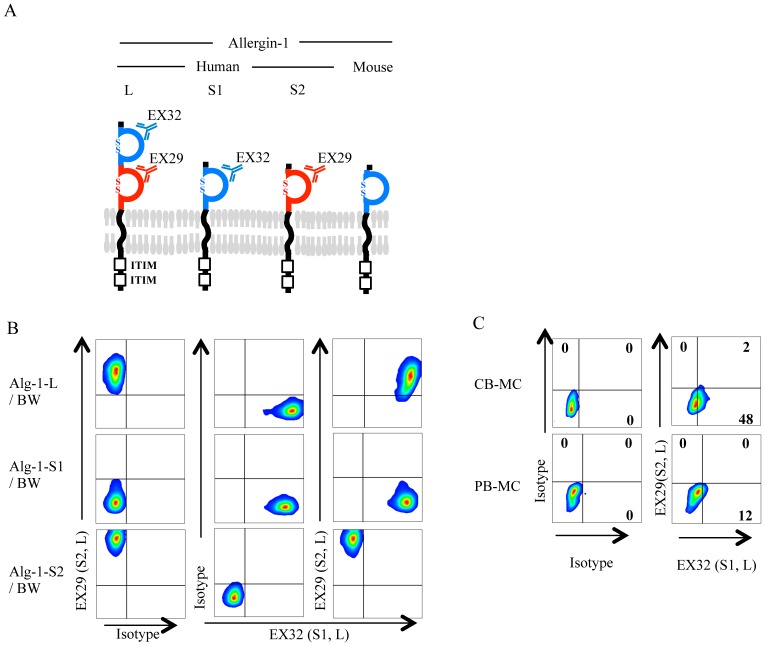
Expression of Allergin-1 isoforms on CB-MC and PB-MC. A. Structure of human and mouse Allergin-1. Unlike mouse Allergin-1, human Allergin-1 consists of three splice isoforms, Allergin-1L, Allergin-1S1, and Allergin-1S2. The first and second immunoglobulin-like domains in the extracellular portion of Allergin-1L is identical to that of Allergin-1S1 and Allergin-1S2, respectively. B. BW5147 transfectants stably expressing each isoform of Allergin-1 were stained with isotype control, anti-Allergin-1S1-specific EX32, and/or anti-Allergin-1S2-specific EX29 mAbs and were analyzed by using flow cytometry. C. CB-MC and PB-MC were stained with isotype control Abs or with EX32 and EX29 mAbs and were analyzed by using flow cytometry.

To examine the protein expression profile of the Allergin-1 isoforms on MC, we generated three mAbs (clones: EX29, EX32, and EX33) against human Allergin-1 (**Fig. 2A**). To examine the specificities of these mAbs, we generated BW5147 transfectants stably expressing Allergin-1L, Allergin-1S1, or Allergin-1S2 [14]. EX32 and EX33 bound to transfectants expressing Allergin-1L or Allergin-1S1, but not Allergin-1S2. In contrast, EX29 bound to transfectant expressing Allergin-1L or Allergin-1S2, but not Allergin-1S1 (**Fig. 2B** **, and data not shown**), indicating that EX32 and EX33 recognize an epitope of the first Ig-like domain of Allergin-1L, and EX29 recognizes an epitope of the second Ig-like domain. By using EX32 and EX29, we examined the expression of the Allergin-1 splicing isoforms on cultured MC (CB-MC and PB-MC). EX32 stained both CB-MC and PB-MC, whereas EX29 did not stain CB-MC or PB-MC (**Fig. 2C**), suggesting that these cultured MC express Allergin-1S1, but not Allergin-1L or Allergin-1S2.

### Isolation and characterization of human primary MC in BALF

**Figure 3:**
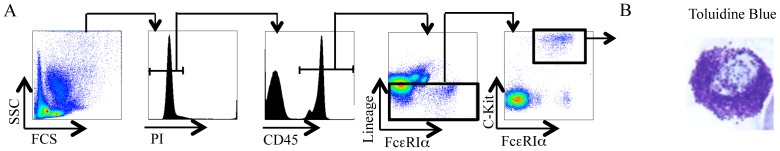
Flow cytometric analyses of BAL-MC. A. BALF cells were simultaneously stained with PI, anti-CD45, a mAb cocktail against lineage markers containing anti-CD3, anti-CD19, anti-CD56, anti-CD11b, and anti-CD11c mAbs, anti-CD117 (c-Kit), and anti-FcεRIα and were analyzed by flow cytometry. B. Bronchoalveolar fluid MC (BAL-MC) sorted by flow cytometry were cytospinned onto glass slides, stained with Toluidine-blue and analyzed by microscopy (×100).

To examine the expression of Allergin-1 isoforms on human primary MC, we first developed a flow cytometric method to characterize the human primary MC in the BALF. We obtained the BALF cells from patients with pulmonary diseases and simultaneously stained the cells with PI and mAbs against CD45; the lineage markers CD3, CD19, CD56, CD11b, and CD11c; c-Kit; and FcεRIα and were analyzed by using multicolor flow cytometry. MC were defined as PI^-^CD45^+^Lin^-^c-Kit^+^ FcεRIα^+^ cells (**Fig. 3A**), which comprised 0.153%±0.041% (*n* = 28) of the total cell population in BALF. No significant differences were observed in the frequency of MC in BALF (BAL-MC) from patients with pulmonary diseases such as idiopathic interstitial pneumonia, sarcoidosis, or eosinophilic pneumonia (**Table S1**). Staining of BAL-MC sorted from BALF with Toluidine-blue showed a characteristics of MC with rich granules in the cytoplasm (**Fig. 3B**).

### Expression of Allergin-1 isoforms on human primary MC in BALF

**Figure 4:**
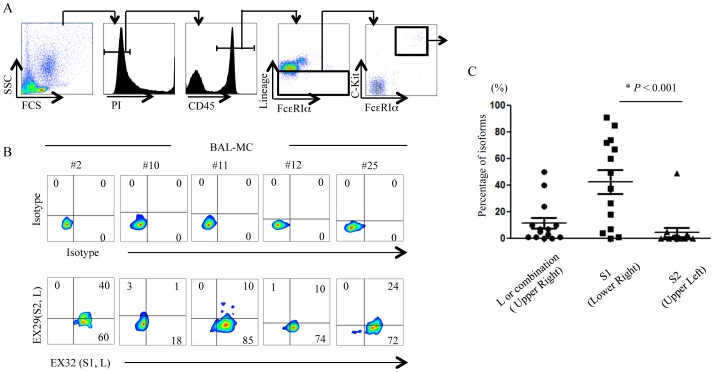
Expression of Allergin-1 isoforms on human BAL-MC. Cells obtained from the BALF of patients with pulmonary diseases were stained with PI, anti-CD45, lineage mAb cocktail, anti-FcεRIα, and anti-c-Kit mAbs together with isotype control, and EX32 and/or EX29 mAb, and the BAL-MC were analyzed by using flow cytometry (A). Representative flow cytometry profiles of five patients are shown (B). “Combinations” indicates MC in the upper right quadrant that express either L+S1, L+S2, L+S1+S2, or S1+S2 (C).

We then conducted a seven-color flow cytometric analysis in which the cells obtained from the BALF of patients with various pulmonary diseases were simultaneously stained with EX32 and EX29, in addition to PI and mAbs against CD45, lineage markers, c-Kit, and FcεRIα·(**Fig. 4A**). We found diverse expression profiles on BAL-MC among the patients (**Fig. 4B**), and, in accordance with the results from PB-MC and CB-MC, Allergin-1S1 was dominantly expressed on BAL-MC, compared with Allergin-1L1 or Allergin-1S2 (**Fig. 4C**).

### Expression of Allergin-1 isoforms on human primary MC in the nasal cavity

**Figure 5:**
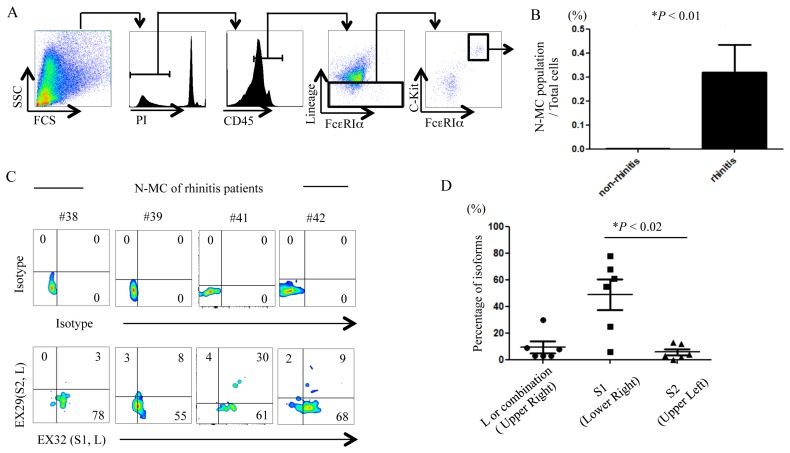
Expression of Allergin-1 isoforms on human N-MC. A, B. NSS cells obtained from 9 patients with allergic rhinitis or 5 healthy donors (non-rhinitis) were stained with PI, anti-CD45, lineage cocktail, anti-FcεRIα, and anti-c-Kit mAbs and analyzed by flow cytometry (A). The frequencies of N-MC population were compared between two groups (B). C, D. NSS cells obtained from 6 patients with allergic rhinitis were stained with mAbs, as described in A, together with EX32 and EX29 mAbs, and analyzed by flow cytometry. Representative flow cytometry profiles of four patients are shown (C). “Combinations” indicates MC in the upper right quadrant that express either L+S1, L+S2, L+S1+S2, or S1+S2 (D).

We also examined MC obtained from the nasal cavity (N-MC) of healthy donors and patients with non-seasonal allergic rhinitis by using multicolor flow cytometry. We detected few N-MC in nasal scratching specimens from healthy donors. In contrast, we clearly observed N-MC in nasal scratching specimens from patients with non-seasonal rhinitis (**Fig. 5A**). The number of N-MC obtained from rhinitis patients was significantly higher than that from healthy donors (**Fig. 5B**). Flow cytometric analysis showed that N-MC preferentially expressed Allergin-1S1, rather than Allergin-1L or Allergin-1S2 (**Fig. 5C, D**), suggesting that Allergin-1S1 is the dominant isoform of Allergin-1 in human primary MC.

### Allergin-1 inhibits IgE-mediated activation of human cultured and primary MC

**Figure 6:**
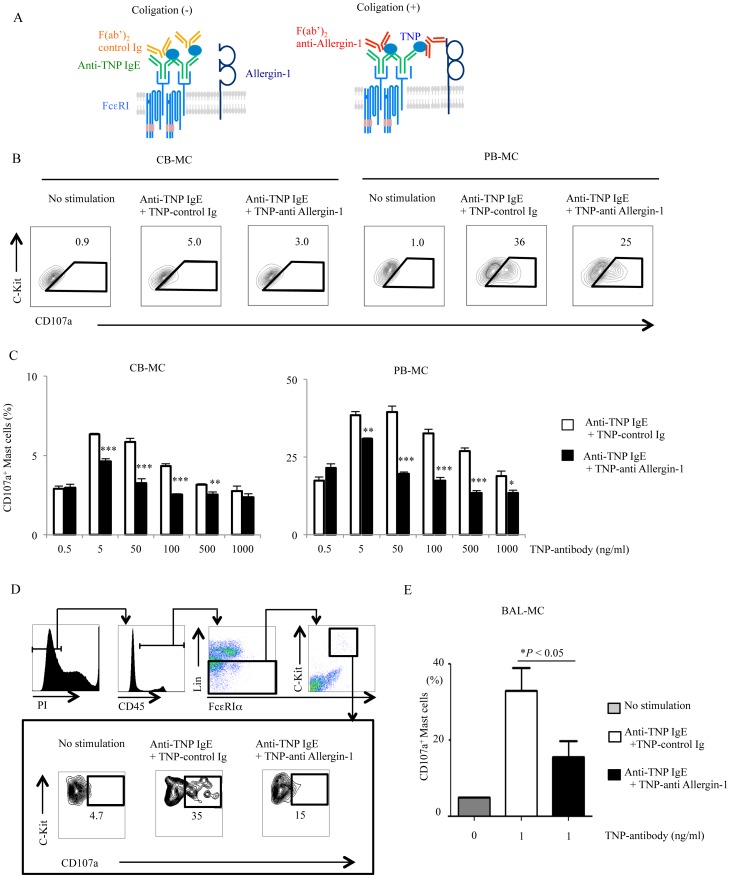
Allergin-1 inhibits IgE-mediated activation of cultured and primary MC. A-C. CB-MC or PB-MC were left unstimulated or stimulated with anti-TNP IgE together with either TNP-conjugated F(ab′)_2_ anti-Allergin-1 (specific to Allergin-1S1) or control Ig, as described in the Methods, and analyzed for the expression of CD107a. D, E. BALF cells were left unstimulated or stimulated, as described in A, and stained with mAbs indicated, as described in the Methods, for MC gating. p<0.05; *, p<0.01; **, p<0.001; ***.

We previously demonstrated that colligation of FcεRIα and Allergin-1 inhibits IgE-mediated activation, as determined by β-hexosaminidase release assay, from an RBL-2H3 transfectant expressing mouse Allergin-1 [14]. However, it remained undetermined whether Allergin-1 also inhibits IgE-mediated activation of human primary MC. Because too small number of human primary MC can be obtained to conduct the ELISA assay for chemical mediators such as histamine or β-hexosaminidase released from MC in of BALF samples, we performed an activation assay by utilizing a multicolor flow cytometry. We first established an activation assay for CB-MC and PB-MC and examined whether Allergin-1 inhibits activation by flow cytometry. CB-MC or PB-MC were incubated with TNP-specific mouse IgE and then the TNP-conjugated F(ab′)_2_ fragment of control Ig or EX33 (anti-Allergin-1L/S1) (**Fig. 6A**). By using cell surface CD107a as a marker of murine and human mast cell degranulation [17]–[19], we detected CB-MC and PB-MC that turned to be positive for CD107a expression on the cell surface when the MC were stimulated with anti-TNP IgE followed by TNP-conjugated control mAb (**Fig. 6B**). However, CD107a^+^ cells were significantly decreased when FcεRIα was colligated with Allergin-1 with anti-TNP IgE and TNP-conjugated anti-Allergin-1 mAb (**Fig. 6B, C**). Since CB-MC and PB-MC preferentially express Allergin-1S1, but neither Allergin-1L nor Allergin-1S2, these results indicated that Allergin-1S1 inhibits IgE-mediated activation from cultured human MC.

We then examined the inhibitory function of Allergin-1 on MC in BALF. BALF cells were also stimulated and then stained with anti-CD107a and mAbs for the identification of BAL-MC, as described above, and analyzed by using multi-color flow cytometry. We detected a subpopulation of BAL-MC that was positive for CD107a when the BALF cells were stimulated with anti-TNP IgE followed by TNP-conjugated control mAb (**Fig. 6D**). However, the population of CD107a^+^ MC were significantly decreased when BAL-MC were stimulated via anti-TNP IgE and TNP-conjugated anti-Allergin-1 mAb (**Fig. 6E**). Since BAL-MC also preferentially express Allergin-1S1 rather than Allergin-1L or Allergin-1S2, these results indicated that Allergin-1S1 inhibits the IgE-mediated activation of BAL-MC.

## Discussion

Previous reports have shown the phenotypical characteristics of human primary MC in bone marrow, lung, skin, intestine, uterus, heart, and ascites by using flow cytometry, in which MC were defined as CD45^+^c-Kit^+^IgE^+^ cells or CD45^+^c-Kit^+^ cells [20], [21]. However, c-Kit is also expressed on hematopoietic progenitors, and recent reports further demonstrate that c-Kit is also expressed on natural helper cells [22], [23]. MC are distinguished from hematopoietic progenitors and natural helper cells by the expression of FcεRI; however, FcεRIα· is also expressed on basophils. In addition, FcεRIα is a component of the FcεRIα trimer expressed on some myeloid lineage cells. Thus, we expected that the co-expression of c-Kit and FcεRIα is not enough for the definition of MC. In this study, we defined human primary MC as PI^-^CD45^+^c-Kit^+^ FcεRIα^+^ cells in the Lin^-^ cell population. Indeed, we found that PI^-^CD45^+^c-Kit^+^ FcεRIα^+^ cells including the Lin^+^ cells contained a significant population of non-MC contaminated (data not shown), thus leading to inaccurate analyses of primary MC.

Previous studies often used conventional histamine, cytokine or β-hexosaminidase release assay by ELISA to investigate activation of purified and enriched MC obtained from the lung or skin in the presence of growth factors and cytokines [19], [24], [25]. However, ELISA can hardly be applied for a small amount of protein produced by a very small number of primary uncultured MC. Our approach can be applied to the functional study of human primary MC, even at the extremely low frequencies seen in BALF. The methodology in this study enable us to show the function of inhibitory receptors on human primary MC for the first time.

By using this method together with the simultaneous application of EX32 and EX29, which are mAbs specific to Allergin-1S1 and Allergin-1S2, respectively, we developed a unique approach to analyzing the expression profile of Allergin-1 isoforms on primary MC in BALF and NSS. We demonstrated that Allergin-1S1 was predominantly expressed on MC in both BALF and NSS. A database search identified mouse and rat Allergin-1 that contained a single Ig-like domain in the extracellular portion with amino acid identities of 50% and 52%, respectively, with that of human Allergin-1S1 [14], suggesting that Allergin-1S1 is well conserved. We previously reported that Allergin-1 expression on MC suppresses the development of IgE- and MC-dependent systemic and cutaneous anaphylaxis in mice [14], suggesting that human Allergin-1S1 may also play an important role in allergic responses. In fact, we demonstrated that the Allergin-1S1–mediated signal suppressed FcεRI-mediated activation in primary MC. However, since Allergin-1 is also expressed on myeloid cells, including monocytes, granulocytes and dendritic cells [14], as well as MC in human, a therapeutic approach for allergic diseases by targeting Allergin-1 may be complex. To develop a novel molecular target therapy by using Allergin-1, the role of Allergin-1 on myeloid cells as well as MC in allergic and non-allergic diseases should be clarified.

In this study, we analyzed the MC in BALF from 28 patients with various pulmonary diseases. We observed no significant differences in the frequency of MC among the patients. Previous reports demonstrated that the frequencies of MC, as determined by microscopic analysis, are much higher in BALF from patients with hypersensitivity pneumonitis [26], [27], cryptogenic organizing pneumonia [27], extrinsic allergic alveolitis [28], interstitial pneumonia [29], [30], or allergic rhinitis and asthma [31] than in healthy controls. We could not compare BAL-MC frequency between patients and healthy controls; however, the frequency of MC in NSS was significantly higher in patients with rhinitis than that in healthy controls, suggesting that recruitment of MC to the nasal mucosa or MC growth within the tissues of the body are augmented in patients with rhinitis.

## Supporting Information

Table S1 **The patients' background.** IIP; idiopathic interstitial pneumonia, CVD-ILD; collagen vascular disease associated interstitial lung disease, BALF; bronchoalveolar lavage fluid, NSS; nasal scratching specimen, *P = 0.0032 compared to control.
(PPTX)

Table S2 **Profile of antibodies used in this study.**
(PPTX)
