# Supplementary material for: Expression and Function of Allergin-1 on Human Primary Mast Cells
Source: PLoS One. 2013 Oct 7;8(10):e76160. doi: 10.1371/journal.pone.0076160 (PMC3792105; doi:10.1371/journal.pone.0076160)
Supplement: Table S1 — The patients' background. IIP; idiopathic interstitial pneumonia, CVD-ILD; collagen vascular disease associated interstitial lung disease, BALF; bronchoalveolar lavage fluid, NSS; nasal scratching specimen, *P = 0.0032 compared to control. (PPTX) [file pone.0076160.s001.pptx]

## Slide 1
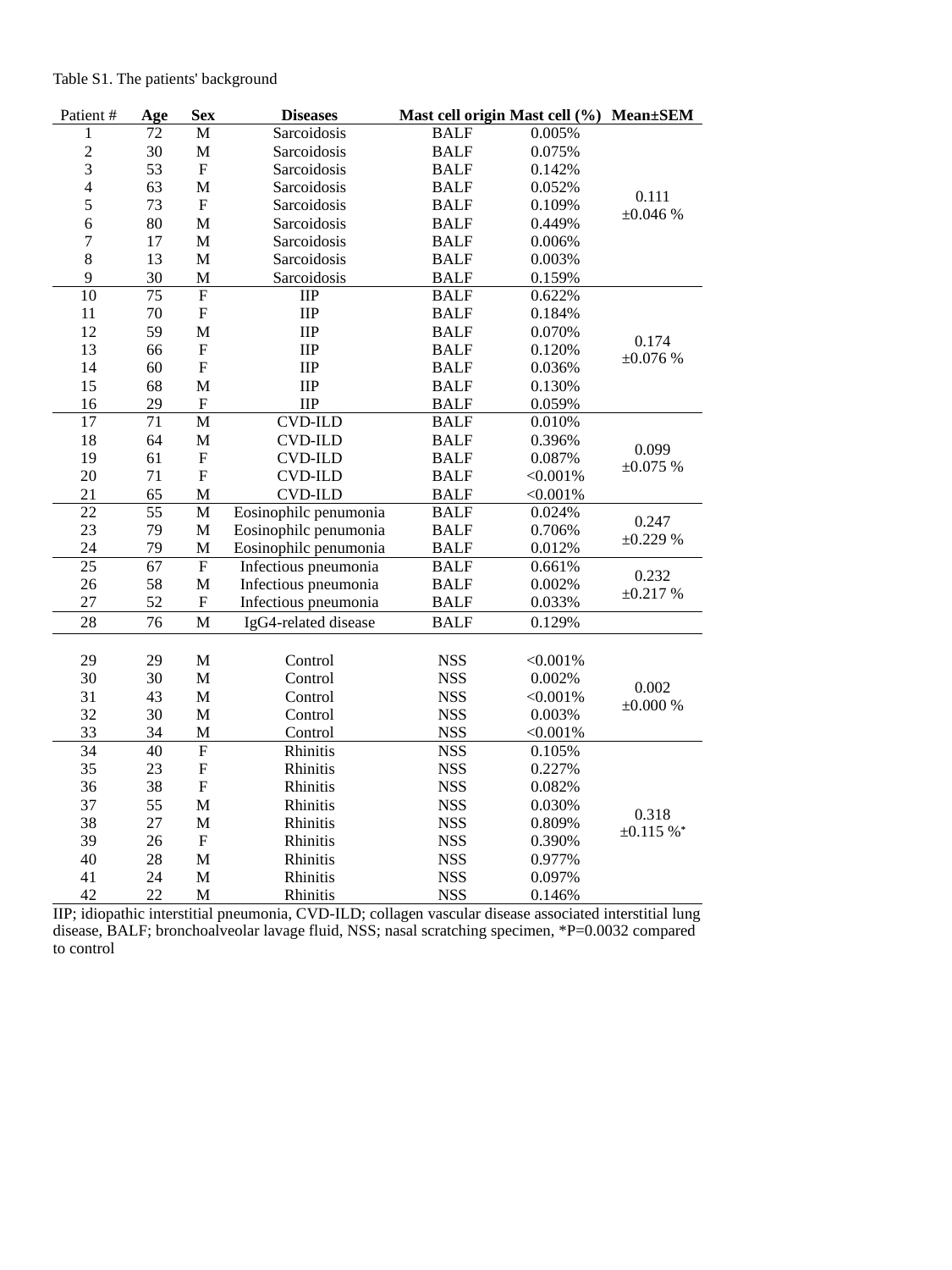

| | | | | | | |
| --- | --- | --- | --- | --- | --- | --- |
| Table S1. The patients' background | | | | | | |
| | | | | | | |
| Patient # | Age | Sex | Diseases | Mast cell origin | Mast cell (%) | Mean±SEM |
| 1 | 72 | M | Sarcoidosis | BALF | 0.005% | 0.111 ±0.046 % |
| 2 | 30 | M | Sarcoidosis | BALF | 0.075% | |
| 3 | 53 | F | Sarcoidosis | BALF | 0.142% | |
| 4 | 63 | M | Sarcoidosis | BALF | 0.052% | |
| 5 | 73 | F | Sarcoidosis | BALF | 0.109% | |
| 6 | 80 | M | Sarcoidosis | BALF | 0.449% | |
| 7 | 17 | M | Sarcoidosis | BALF | 0.006% | |
| 8 | 13 | M | Sarcoidosis | BALF | 0.003% | |
| 9 | 30 | M | Sarcoidosis | BALF | 0.159% | |
| 10 | 75 | F | IIP | BALF | 0.622% | 0.174 ±0.076 % |
| 11 | 70 | F | IIP | BALF | 0.184% | |
| 12 | 59 | M | IIP | BALF | 0.070% | |
| 13 | 66 | F | IIP | BALF | 0.120% | |
| 14 | 60 | F | IIP | BALF | 0.036% | |
| 15 | 68 | M | IIP | BALF | 0.130% | |
| 16 | 29 | F | IIP | BALF | 0.059% | |
| 17 | 71 | M | CVD-ILD | BALF | 0.010% | 0.099 ±0.075 % |
| 18 | 64 | M | CVD-ILD | BALF | 0.396% | |
| 19 | 61 | F | CVD-ILD | BALF | 0.087% | |
| 20 | 71 | F | CVD-ILD | BALF | <0.001% | |
| 21 | 65 | M | CVD-ILD | BALF | <0.001% | |
| 22 | 55 | M | Eosinophilc penumonia | BALF | 0.024% | 0.247 ±0.229 % |
| 23 | 79 | M | Eosinophilc penumonia | BALF | 0.706% | |
| 24 | 79 | M | Eosinophilc penumonia | BALF | 0.012% | |
| 25 | 67 | F | Infectious pneumonia | BALF | 0.661% | 0.232 ±0.217 % |
| 26 | 58 | M | Infectious pneumonia | BALF | 0.002% | |
| 27 | 52 | F | Infectious pneumonia | BALF | 0.033% | |
| 28 | 76 | M | IgG4-related disease | BALF | 0.129% | |
| | | | | | | |
| 29 | 29 | M | Control | NSS | <0.001% | 0.002 ±0.000 % |
| 30 | 30 | M | Control | NSS | 0.002% | |
| 31 | 43 | M | Control | NSS | <0.001% | |
| 32 | 30 | M | Control | NSS | 0.003% | |
| 33 | 34 | M | Control | NSS | <0.001% | |
| 34 | 40 | F | Rhinitis | NSS | 0.105% | 0.318 ±0.115 %\* |
| 35 | 23 | F | Rhinitis | NSS | 0.227% | |
| 36 | 38 | F | Rhinitis | NSS | 0.082% | |
| 37 | 55 | M | Rhinitis | NSS | 0.030% | |
| 38 | 27 | M | Rhinitis | NSS | 0.809% | |
| 39 | 26 | F | Rhinitis | NSS | 0.390% | |
| 40 | 28 | M | Rhinitis | NSS | 0.977% | |
| 41 | 24 | M | Rhinitis | NSS | 0.097% | |
| 42 | 22 | M | Rhinitis | NSS | 0.146% | |
| IIP; idiopathic interstitial pneumonia, CVD-ILD; collagen vascular disease associated interstitial lung disease, BALF; bronchoalveolar lavage fluid, NSS; nasal scratching specimen, \*P=0.0032 compared to control | | | | | | |
| | | | | | | |
