# Supplementary material for: Expression and Function of Allergin-1 on Human Primary Mast Cells
Source: PLoS One. 2013 Oct 7;8(10):e76160. doi: 10.1371/journal.pone.0076160 (PMC3792105; doi:10.1371/journal.pone.0076160)
Supplement: Table S2 — Profile of antibodies used in this study. (PPTX) [file pone.0076160.s002.pptx]

## Slide 1
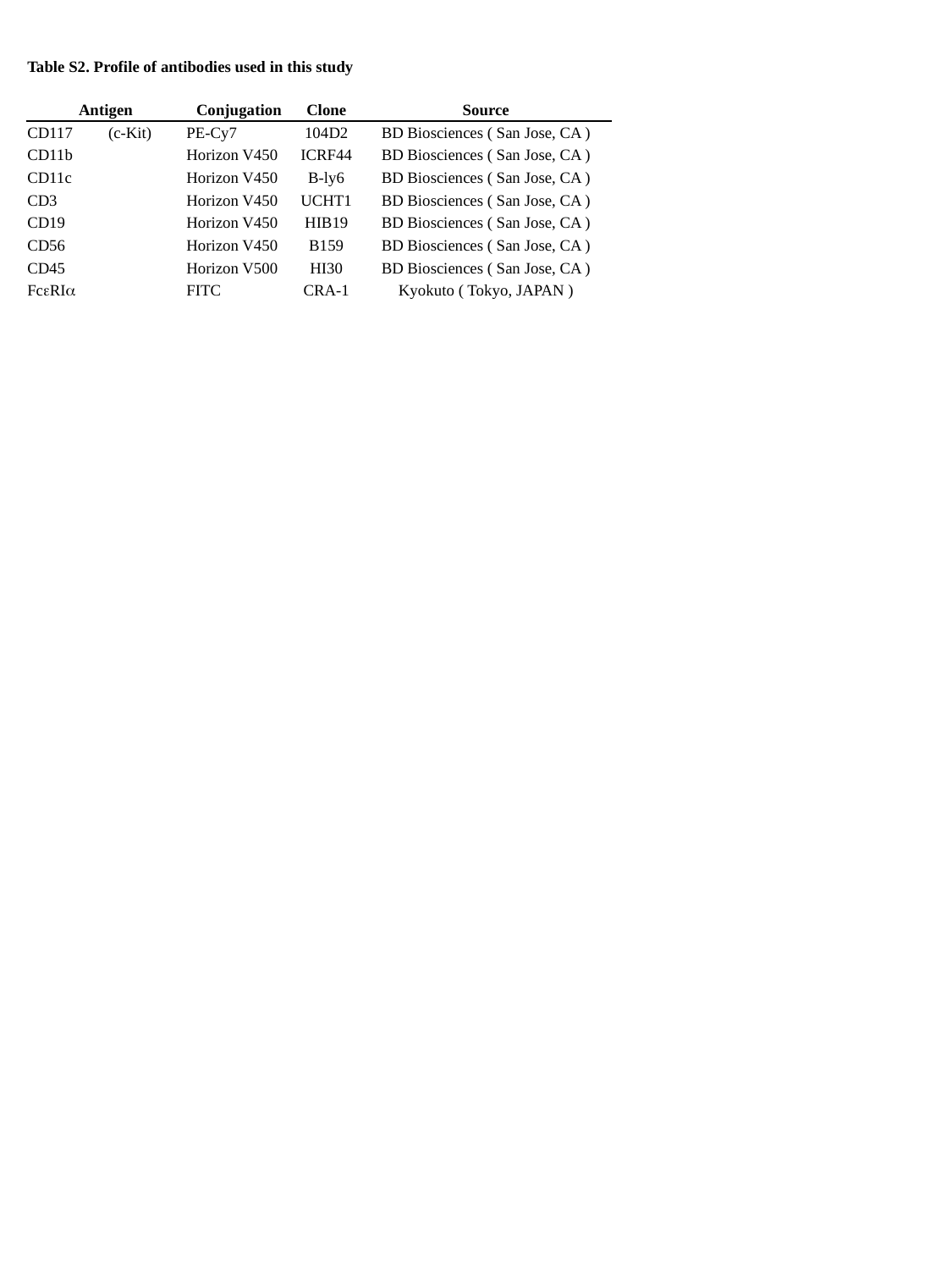

| | | | | |
| --- | --- | --- | --- | --- |
| Table S2. Profile of antibodies used in this study | | | | |
| | | | | |
| Antigen | | Conjugation | Clone | Source |
| CD117 | (c-Kit) | PE-Cy7 | 104D2 | BD Biosciences ( San Jose, CA ) |
| CD11b | | Horizon V450 | ICRF44 | BD Biosciences ( San Jose, CA ) |
| CD11c | | Horizon V450 | B-ly6 | BD Biosciences ( San Jose, CA ) |
| CD3 | | Horizon V450 | UCHT1 | BD Biosciences ( San Jose, CA ) |
| CD19 | | Horizon V450 | HIB19 | BD Biosciences ( San Jose, CA ) |
| CD56 | | Horizon V450 | B159 | BD Biosciences ( San Jose, CA ) |
| CD45 | | Horizon V500 | HI30 | BD Biosciences ( San Jose, CA ) |
| FceRIa | | FITC | CRA-1 | Kyokuto ( Tokyo, JAPAN ) |
| | | | | |
